# Optogenetic Inhibition of Striatal Parvalbuminergic Interneurons Unmasks Impaired GABA and Adenosine Signaling in DYT1 Knock-In Mice

**DOI:** 10.3390/ijms27104530

**Published:** 2026-05-18

**Authors:** Jakob Marx, Susen Becker, Lisa Höfert, Ina Hochheim, Christin Helmschrodt, Jan Dreßler, Angelika Richter, Anja Schulz

**Affiliations:** 1 Institute of Pharmacology, Pharmacy and Toxicology, Faculty of Veterinary Medicine, University of Leipzig, 04103 Leipzig, Germany; hochheim@vetmed.uni-leipzig.de (I.H.); christin.helmschrodt@vetmed.uni-leipzig.de (C.H.); angelika.richter@vetmed.uni-leipzig.de (A.R.);; 2 Institute of Legal Medicine, Medical Faculty, University of Leipzig, 04103 Leipzig, Germany; susen.becker@medizin.uni-leipzig.de (S.B.); lisa.hoefert@medizin.uni-leipzig.de (L.H.); jan.dressler@medizin.uni-leipzig.de (J.D.)

**Keywords:** optodialysis, microdialysis, optogenetics, fast-spiking interneurons, GABA, adenosine, DYT1 dystonia

## Abstract

Neurochemical imbalances in the striatum are thought to contribute to the pathophysiology of DYT1 dystonia (TOR1A), a severe movement disorder. Parvalbumin-positive GABAergic fast-spiking interneurons (PV+ FSI) exert a powerful inhibition within the striatal microcircuitry. To elucidate the impact of PV+ FSI on striatal neurotransmitter dynamics in a DYT1 knock-in (KI) mouse model, we combined optogenetic inhibition of PV+ FSI with in vivo microdialysis (optodialysis) and LC-MS/MS analysis. Dialysates were collected across baseline (light off), stimulation (light on, 595 nm), and post-stimulation (light off) periods. Basal extracellular concentrations of several analytes, including GABA, dopamine, and adenosine, showed no significant differences between wild-type (WT) and DYT1 KI mice. In WT mice, PV+ FSI inhibition decreased GABA and adenosine levels. In contrast, DYT1 KI mice showed no change in GABA and only a delayed reduction in adenosine post-stimulation. Dopamine, choline, or 5-HIAA were largely unaffected by optogenetic inhibition, with the exception of a genotype-specific reduction of 5-HIAA in the post-stimulation period. These findings suggest impaired inhibitory and neuromodulatory control in the DYT1 KI mice, potentially reflecting compensatory circuit adaptations. The results provide novel insights into striatal microcircuit function in DYT1 dystonia, establish a basis for exploring circuit-level alterations in other movement disorders, and may inform future therapeutic strategies.

## 1. Introduction

Dystonia is a movement disorder characterized by involuntary, sustained, or intermittent muscle contractions resulting in abnormal, often repetitive, movements and postures [1]. One hereditary form, DYT1 dystonia, is an early-onset, generalized, autosomal dominant disorder with incomplete penetrance, affecting approximately 30% of mutation carriers [2]. A base pair deletion (ΔGAG) in the TOR1A gene causes a pathogenic codon deletion (ΔE) in the ATPase TorsinA, which impairs vesicle transport, protein folding, and neurotransmitter release. Although ΔE-TorsinA contributes to dystonia pathophysiology, the underlying mechanisms remain unclear [3]. The early postnatal lethality in homozygous DYT1 knock-in (KI) mice supports a loss-of-function mutation [4].

Alterations in striatal GABAergic, dopaminergic, and cholinergic pathways have been reported in various dystonia models, suggesting a central role in disease pathophysiology [5]. In heterozygous DYT1 (KI) mice, a mouse model of asymptomatic human mutation carriers, subtle sensorimotor deficits and signs of aberrant synaptic plasticity have been described [6,7,8,9]. Furthermore, these mice exhibit reduced basal levels of dopamine (DA) [7,10], impaired DA release [7,10,11], and elevated extracellular acetylcholine (ACh) [12].

GABAergic parvalbumin-positive fast-spiking interneurons (PV+ FSI) are crucial regulators of striatal inhibition, providing strong feedforward inhibition onto striatal projection neurons (SPN) and interacting with GABAergic, dopaminergic, and cholinergic systems [13,14]. Reduced inhibitory control in the striatum, along with PV+ FSI dysfunction, is considered to contribute to the pathophysiology of dystonia [15,16,17,18,19,20,21]. Recent work demonstrated that in vivo optogenetic inhibition of PV+ FSI did not induce dystonic or dyskinetic signs, but caused a genotype-specific decrease in neuronal activity, as indicated by less c-Fos immunoreactivity, in DYT1 KI mice [19]. Despite their critical role, the contribution of PV+ FSI to extracellular striatal neurotransmitter regulation remains unclear.

To address this gap, we used optodialysis—a combination of in vivo microdialysis (MD) and optogenetics—to modulate neuronal activity [22] and monitor extracellular neurotransmitters in freely moving mice [23]. We simultaneously measured striatal extracellular levels of γ-aminobutyric acid (GABA), dopamine (DA), 3-methoxytyramine (3-MT), acetylcholine (ACh) and its isobaric structure iso-acetylcholine (isoACh), choline, adenosine (ADE), and 5-hydroxyindolacetic acid (5-HIAA) [24,25] across baseline, light stimulation, and post-stimulation periods. This approach was designed to reveal how PV^+^ FSI inhibition shapes striatal neurotransmitter dynamics and whether these dynamics are altered in the DYT1 KI genotype. Establishing optodialysis as a viable tool for circuit-specific neurochemical profiling may furthermore serve as a methodological foundation for investigating other genetic movement disorders.

## 2. Results

### 2.1. Verifying Localization and Expression

**Figure 1:**
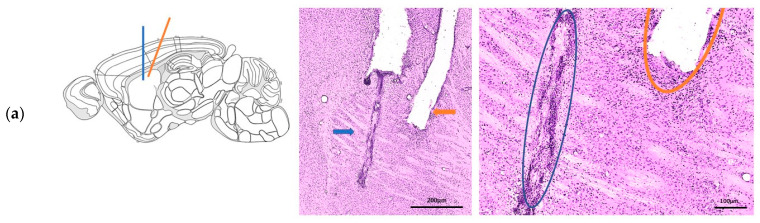

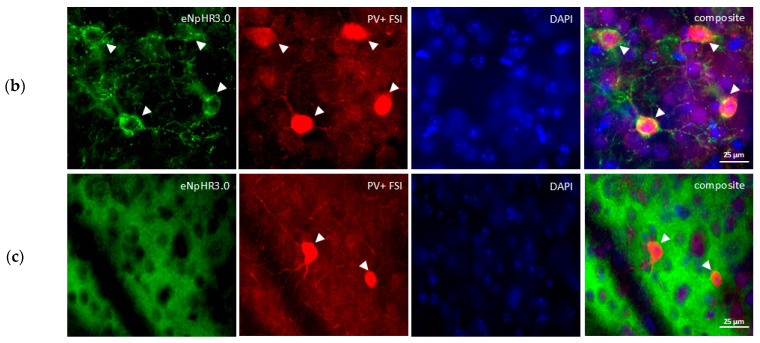
(**a**) **Left:** Schematic illustration of implantation into the dorsal striatum. **Middle**: Verification of implant localization and cell-type-specific halorhodopsin (eNpHR3.0) expression in PV+ FSI Nissl-stained coronal section of microdialysis (MD) guide cannula (blue arrow and circle) and optical fiber (orange arrow and circle) within the dorsal striatum. The maximum spacing (≤200 µm) ensured sampling within the optical range; scale bar 200 µm; **Right**: scale bar 100 µm. (**b**) Specific expression of eNpHR3.0 (green) in PV+ FSI (red); cell nuclei stained with DAPI (blue), scale bar 25 µm. (**c**) Non-specific expression of eNpHR3.0 in PV+ FSI, representing the exclusion criterion, scale bar 25 µm.

Placement of the MD probe and the optical fiber within the dorsal striatum was verified in all experimental animals using Nissl staining (Figure 1a). Only mice with a strong local tropism of eNpHR3.0 expression in PV+ FSI were included in the final analysis (Figure 1b). WT and two DYT1 KI mice were excluded due to non-specific eNpHR3.0 expression beyond PV+ FSI (Figure 1c).

### 2.2. Extracellular Neurotransmitter and Neuromodulator Levels

Extracellular baseline concentrations of all analyzed neurotransmitters and neuromodulators—including dopamine (DA), 3-methoxytyramine (3-MT), iso-acetylcholine (isoACh), γ-aminobutyric acid (GABA), acetylcholine (ACh), choline, 5-hydroxyindolacetic acid (5-HIAA), and adenosine (ADE)—did not differ significantly between WT and DYT1 KI mice. A full dataset is provided in the Supplementary Materials (Tables S1 and S2). The absence of baseline differences between genotypes indicates that the tonic neurochemical tone is preserved in DYT1 KI mice under resting conditions, and that any genotype-specific effects are attributable to stimulus-evoked dynamics rather than pre-existing concentration differences.

Table 1 summarizes the mean concentrations of all analytes across conditions.

#### 2.2.1. GABA Levels Decreased upon Optogenetic Inhibition of PV+ FSI in WT but Not in DYT1 KI

**Figure 2:**
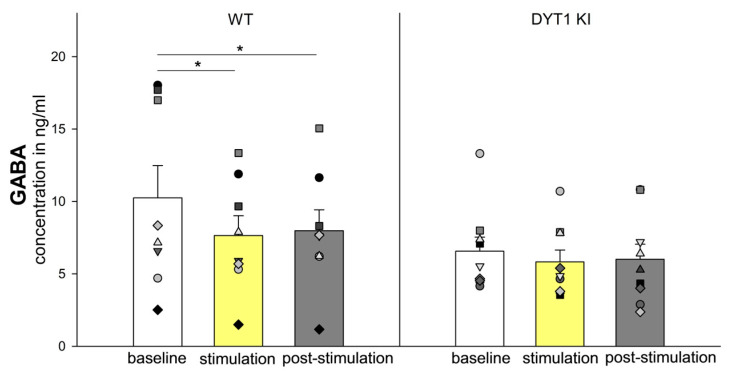
Optogenetic inhibition of PV+ FSI decreases GABA levels in WT but not in DYT1 KI mice. The bars show the group means ± SEM of extracellular GABA concentration and the scatter plots show individual mouse data as repeated measurements from the same animal across conditions (each symbol represents one animal). WT *n* = 8, DYT1 KI *n* = 9, * WT, baseline vs. stimulation, *p* < 0.05, vs. post-stimulation, *p* < 0.05 (Holm Sidak).

Although GABA levels tended to be lower in DYT1 KI mice than in WT, no significant differences were detected [F (1/50) = 1.949, *p* = 0.183] (Figure 2). However, optogenetic stimulation significantly affected GABA levels [F (2/50) = 4.829, *p* = 0.015]. Post hoc analysis revealed a significant decrease in GABA levels in WT mice (*n* = 8) during stimulation (baseline 10.25 ± 2.23 ng/mL vs. stimulation 7.64 ± 1.37 ng/mL, *p* = 0.013) and post-stimulation (baseline vs. post-stimulation 7.98 ± 1.44 ng/mL, *p* = 0.023). GABA levels in DYT1 KI mice remained relatively stable across all stimulation periods (*n* = 9, baseline 6.57 ± 0.97 ng/mL vs. stimulation 5.82 ± 0.82 ng/mL vs. post-stimulation 6.01 ± 1.04 ng/mL).

#### 2.2.2. DA and 3-MT Levels Remained Stable Across Stimulation

**Figure 3:**
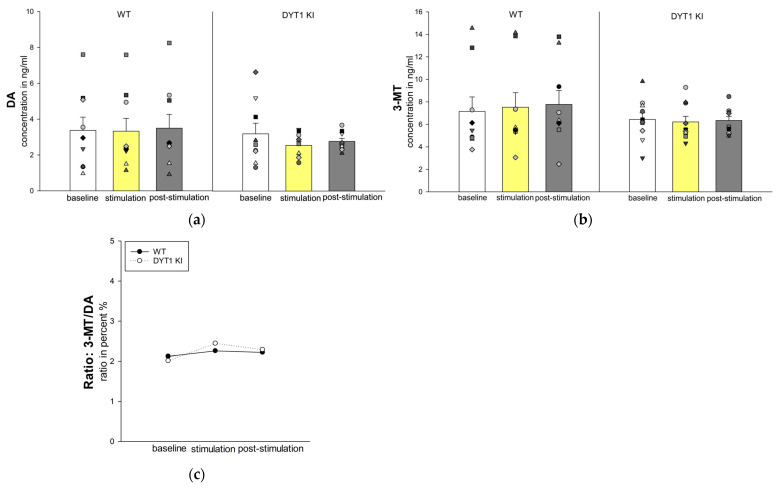
Dopamine and 3-MT were unaffected by optogenetic inhibition of PV+ FSI in both genotype concentrations in WT and DYT1 KI mice. The bars show the group means ± SEM of extracellular DA (**a**) and 3-MT (**b**) concentration and the scatter plots show individual mouse data as repeated measurements from the same animal across conditions (each symbol represents one animal). (**c**) Ratio of 3-MT/DA in WT and DYT1 KI. WT *n* = 9/9, DYT KI *n* = 9/10 (DA/3-MT).

DA levels showed no significant differences between genotypes or across stimulation periods (Figure 3a). In WT mice, DA concentrations remained stable (*n* = 9, baseline 3.37 ± 0.74 ng/mL, stimulation 3.33 ± 0.71 ng/mL, post-stimulation 3.50 ± 0.76 ng/mL). In DYT1 KI mice, DA concentrations decreased slightly during stimulation and recovered moderately post-stimulation (*n* = 9, baseline 3.19 ± 0.59 ng/mL, stimulation 2.54 ± 0.23 ng/mL, post-stimulation 2.77 ± 0.17 ng/mL). However, these changes were not statistically significant. Similarly, the concentration of the DA metabolite 3-MT remained unchanged across all conditions (WT *n* = 9 vs. DYT1 KI *n* = 10, baseline: 7.15 ± 1.29 ng/mL vs. 6.43 ± 0.59 ng/mL, stimulation: 7.52 ± 1.29 ng/mL vs. 6.12 ± 0.51 ng/mL, post-stimulation: 7.77 ± 1.24 ng/mL vs. 6.34 ± 0.37 ng/mL; Figure 3b).

The 3-MT/DA, reflecting extracellular dopamine turnover, did not differ significantly between genotypes or across conditions, suggesting that DA metabolism was unaffected by PV+ FSI.

The stability of both DA and 3-MT levels across conditions and genotypes indicates that optogenetic inhibition of PV+ FSI did not engage dopaminergic pathways, underscoring the selectivity of PV+ FSI inhibition for GABAergic transmission and adenosine-mediated neuromodulation (Figure 3c).

#### 2.2.3. Extracellular Adenosine (ADE) Levels Decreased with a Delay During Inhibition of PV+ FSI in DYT1 KI

**Figure 4:**
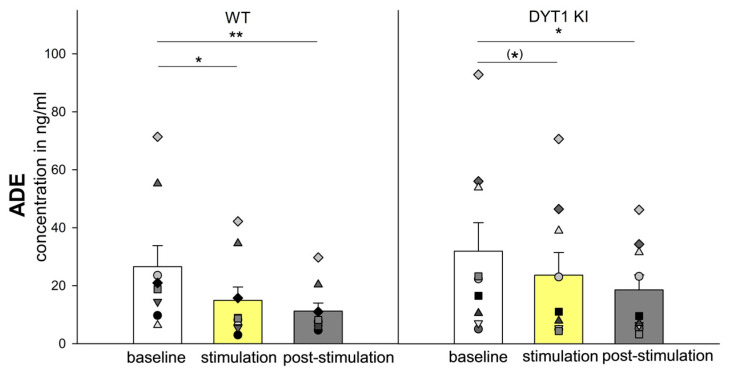
Optogenetic inhibition of PV+ FSI decreased adenosine levels immediately in WT but with a delay in DYT1 KI mice. The bars show the group means ± SEM and the scatter plots show individual mouse data as repeated measurements from the same animal across conditions (each symbol represents one animal). WT *n* = 9, DYT1 KI *n* = 9, * WT, baseline vs. stimulation, *p* < 0.01 (Holm Sidak), ** WT, baseline vs. post stimulation, *p* < 0.001 (Holm Sidak), * DYT1 KI, baseline vs. post stimulation, *p* < 0.01 (Holm Sidak) (*) a trend within DYT1 KI, baseline vs. stimulation, *p* = 0.054 (Holm Sidak).

ADE levels did not significantly differ between genotypes [F (1/53) = 0.631, *p* = 0.439]. A significant main effect of stimulation was detected [F (2/53) = 16.799, *p* < 0.001]. Post-hoc tests revealed a decrease during stimulation and post-stimulation in WT mice (*n* = 9, baseline 26.58 ± 7.30 ng/mL vs. stimulation 14.90 ± 4.63 ng/mL, *p* = 0.005; baseline vs. post-stimulation 11.23 ± 2.79 ng/mL, *p* < 0.001). In DYT1 KI mice, a significant ADE decrease was only observed between baseline and post stimulation period (*n* = 9, baseline 31.96 ± 9.85 ng/mL vs. post stimulation 18.54 ± 5.25 ng/mL, *p* = 0.002), with a statistical trend during stimulation (baseline vs. stimulation 23.64 ± 7.84 ng/mL, *p* = 0.054) (Figure 4).

#### 2.2.4. 5-HIAA Levels Were Selectively Reduced in DYT1 KI Mice During and After Optogenetic Inhibition of PV+ FSI

**Figure 5:**
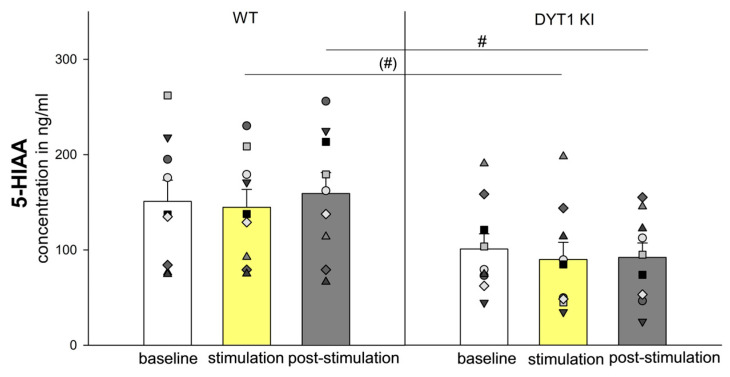
5-HIAA levels were selectively reduced during and after optogenetic inhibition of PV+ FSI. The bars show the group means ± SEM and the scatter plots show individual mouse data as repeated measurements from the same animal across conditions (each symbol represents one animal). WT *n* = 9, DYT1 KI *n* = 9, ^(#)^ within stimulation, WT vs. DYT1 KI, *p* = 0.053 (Holm Sidak), ^#^ within post-stimulation, WT vs. DYT1 KI, *p* = 0.021 (Holm Sidak).

Serotonin (5-HT) was below the limit of detection (< LOD), but its metabolite 5-hydroxyindolacetic acid (5-HIAA) was reliably quantified. A genotype effect was found for 5-HIAA levels [F (1/53) = 5.221, *p* = 0.036], with DYT1 KI mice exhibiting lower concentrations in WT than in DYT1 KI during post-stimulation (WT *n* = 9 vs. DYT1 KI *n* = 9, post-stimulation: 159.15 ± 21.91 ng/mL vs. 91.99 ± 15.19 ng/mL, *p* = 0.021) and tended to be significant during stimulation (WT vs. DYT1 KI, stimulation: 144.65 ± 18.79 ng/mL vs. 89.70 ± 18.10 ng/mL, *p* = 0.053) (Figure 5).

#### 2.2.5. ACh Levels Were Unaffected by Optogenetic Inhibition, but Choline Decreased Post-Stimulation in Both Genotypes

**Figure 6:**
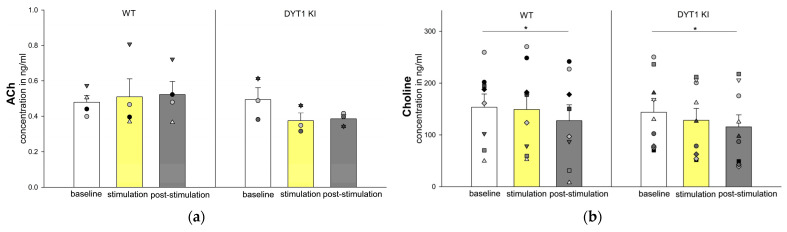
Acetylcholine and Choline concentrations in WT and DYT1 KI mice. The bars show the group means ± SEM of extracellular ACh (**a**) and Choline (**b**) concentration and the scatter plots show individual mouse data representing repeated measurements from the same animal across conditions (each symbol represents one animal). ACh: WT *n* = 4, DYT KI *n* = 3; Choline: WT *n* = 8, DYT1 KI *n* = 9. * WT, baseline vs. post-stimulation, *p* = 0.04 (Holm Sidak), * DYT1 KI, baseline vs. post-stimulation, *p* = 0.015 (Holm Sidak).

Acetylcholine (ACh) levels were quantifiable above the limit of quantification (LOQ) in only a subset of animals (WT *n* = 4, DYT1 KI *n* = 3), limiting statistical power. No significant stimulation or genotype effects were found (Figure 6a). While ACh levels remained stable in WT mice, they appeared lower in DYT1 KI during and after stimulation, though without statistical significance.

In contrast, choline concentrations showed a main effect for stimulation [F (2/50) = 8.186, *p* = 0.001], with a significant decrease from baseline to post-stimulation in WT (*n* = 8, base-line 153.36 ± 25.64 vs. post-stimulation 127.51 ± 30.52, *p* = 0.04) and DYT1 KI mice (*n* = 9, baseline 143.68 ± 23.01 vs. post-stimulation 115.50 ± 23:28, *p* = 0.015) (Figure 6b).

#### 2.2.6. Iso-Acetylcholine (isoACh) Tended to Be Lower in DYT1 KI

**Figure 7:**
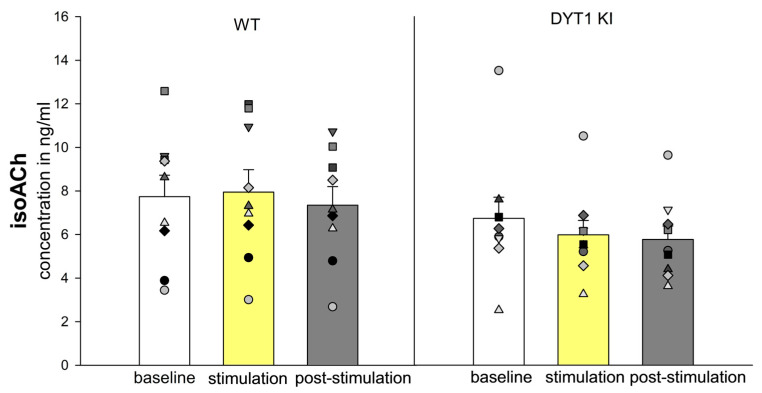
Iso-Acetylcholine concentrations in WT and DYT1 KI mice. The bars show the group means ± SEM and the scatter plots show individual mouse data as repeated measurements from the same animal across conditions (each symbol represents one animal). WT *n* = 9, DYT1 KI *n* = 9.

IsoACh, despite being isobaric with ACh, was chromatographically resolved from ACh using the method described by Becker et al. [25]. No significant genotype or stimulation effect was detected, though a tendency towards reduced post-stimulation levels in DYT1 KI mice was observed (*n* = 9, baseline 6.73 ± 0.97 ng/mL vs. post stimulation 5.77 ± 0.62 ng/mL, *p* = 0.079) (Figure 7). The ACh/isoACh ratio could not be reliably calculated due to the limited data on ACh concentration.

## 3. Discussion

In this study, we combined optogenetic inhibition of parvalbumin-positive fast-spiking interneurons (PV+ FSI) with in vivo microdialysis to investigate striatal neurotransmitter dynamics in a DYT1 dystonia mouse model. Previous work demonstrated changes in neuronal activity following PV+ FSI inhibition [19], but its impact on extracellular neurotransmitter levels remained elusive. Our results reveal genotype-specific differences in response to PV+ FSI inhibition between DYT1 knock-in (KI) mice and wildtype littermates (WT), particularly affecting GABA and adenosine signaling. These results suggest altered inhibitory control and neuromodulatory imbalance in DYT1 KI mice.

### 3.1. Genotypic GABAergic Response to PV+ FSI Inhibition

Optogenetic inhibition of PV+ FSI significantly reduced extracellular GABA levels in WT mice. In contrast, GABA levels remained unchanged in DYT1 KI mice, indicating a genotype-specific disruption of striatal inhibitory signaling. Under physiological conditions, PV+ FSI exert strong inhibition onto both D1- and D2-type striatal projection neurons (SPN), and their widespread axonal arborizations make them major contributors to extracellular GABA [26,27]. Accordingly, optogenetic silencing of PV+ FSI is expected to reduce action potential-dependent GABA release, thereby lowering ambient GABA, as observed in WT mice. While such inhibition may disinhibit SPN, the resulting increase in SPN activity is unlikely to compensate, as they release GABA in a synapse-specific manner and contribute minimally to extracellular GABA [27,28]. However, SPNs are not the sole source of extracellular GABA; other contributors include low-threshold-spiking (LTS) interneurons, SPN collaterals, DA axon co-release, and the regulation of astrocytic GABA uptake [29,30]. The lack of reduced GABA levels in DYT1 KI mice may indicate a ceiling effect from pre-existing GABAergic deficits in dystonia [16], or a structural disconnection resulting from impaired synaptic integration, possibly caused by torsinA-related dysfunction in vesicle trafficking and synaptic organization [31,32]. Consequently, further optogenetic inhibition of PV+ FSI induces only a minimal additional decrease in extracellular GABA levels in DYT1 mice. Unchanged GABA levels in DYT1 KI mice likely reflect compensatory mechanisms: other striatal interneurons, such as somatostatin-positive (SOM+) or LTS interneurons, may be upregulated to maintain inhibitory tone in the striatum [28,33]. Furthermore, recurrent SPN collaterals could contribute to tonic inhibition [34], as suggested in DYT1 models [27,35,36]. Taken together, network-level plasticity in DYT1 dystonia has the potential to reduce network dependence on PV+ FSI activity and buffer against GABA decreases through compensatory mechanisms [9,32,37].

### 3.2. Altered Adenosine Response in DYT1 KI Mice

Adenosine (ADE) levels exhibited genotype-dependent dynamics during PV+ FSI inhibition, with WT mice showing a rapid decline, whereas in DYT1 KI mice, the decrease was both delayed and attenuated. Inhibition of PV+ FSI reduces GABA output onto striatal microcircuits, changing network activity and reducing signals that typically drive ADE accumulation. Since ADE levels strongly reflect neuronal and synaptic activity, a reduction in GABA-driven network activation can either decrease ADE release or increase its clearance rate [38,39]. This framework provides an explanation for the rapid ADE decrease observed in WT mice during PV+ FSI inhibition: the sudden loss of PV+ FSI-mediated inhibition disrupts the balance of synaptic and metabolic activity in striatal circuits, reducing substrate availability for adenosine formation (e.g., ATP breakdown) and potentially enhancing uptake or degradation processes via adenosine kinase or ectonucleoside transporters [40,41,42]. These mechanisms are consistent with established models in which adenosine levels decrease when local neuronal activity diminishes, particularly during reduced inhibitory or excitatory drive [43]. This further underscores the notion that alterations in GABAergic activity directly impact adenosine dynamics.

The genotype-specific differences in ADE levels may reflect disrupted neuromodulatory feedback control in DYT1 dystonia. Several mechanisms could contribute to this, including: (1) reduced adenosine kinase activity [44], (2) increased ectonucleotidase-mediated clearance [45], or (3) dysregulated neurotransmitter crosstalk, particularly involving GABA and DA signaling [12,46]. Notably, an upregulation of adenosine A2A receptors (A2AR) has been described in a DYT1 knock-out model [47]. Although our study used a knock-in model, similar A2AR adaptations could disturb ADE-DA interactions and broader neuromodulatory signaling.

The delayed ADE decrease in DYT1 KI mice aligns with the hypothesis that dystonic circuits fail to appropriately adjust neuromodulatory signals when inhibitory input is perturbed, resulting in blunted feedback inhibition and potentially contributing to sustained hyperexcitability [48]. The dissociation between GABA and ADE dynamics in DYT1 KI mice, where neither neurotransmitter decreases during PV+ FSI inhibition, points toward a more widespread impairment of inhibitory and neuromodulatory feedback mechanisms in the striatum. The present findings of altered GABA and ADE dynamics following PV+ FSI inhibition raise the possibility that these changes contribute to the previously reported synaptic plasticity deficits in DYT1 KI mice [9,32]. However, there is a lack of direct evidence to establish a causal relationship between the observed neurochemical shifts and the alterations in long-term potentiation and long-term depression (LTP/LTD). This is a crucial gap in the existing body of knowledge that should be addressed in future research.

### 3.3. Dopaminergic System: Subtle Shifts, No Significant Effects

In contrast to previous findings in mouse models of DYT1 dystonia, including reduced baseline levels of dopamine (DA) and impaired DA release [7,10,11,49,50], our data show no significant changes in extracellular DA or its metabolite 3-methoxytyramine (3-MT), either at baseline or following PV+ FSI inhibition. Some dopaminergic deficits in DYT1 models occurred only after pharmacological provocation, which was not implemented in this study. Nevertheless, our results differ from the reported ~40% reduction in baseline DA levels in DYT1 KI mice using a comparable in vivo microdialysis (MD) approach [7,10]. Methodological differences, including MD probe placement within striatal regions and animal age or genetic background, could account for these discrepancies [23,51,52].

GABAergic signaling regulates DA release via GABA_A_ and GABA_B_ receptors on dopaminergic terminals [53]. Thus, optogenetic inhibition of PV+ FSI would be expected to disinhibit DA axons and potentially enhance DA release. However, we did not observe an effect. This suggests that GABA derived specifically from PV+ FSI exerts limited influence over dopaminergic terminals in the striatum. Moreover, microdialysis primarily detects tonic, not phasic changes in DA, therefore, short-term silencing of PV+ FSI may produce only subtle, transient changes in extracellular DA [30,53].

Consistent with the absence of DA changes, 3-MT levels were unchanged in both genotypes, aligning with previous findings [17]. A slight, non-significant increase in the 3-MT/DA ratio was observed in DYT1 KI mice during PV+ FSI inhibition. Because 3-MT is formed from extracellular DA via catechol-O-methyltransferase (COMT), this subtle shift may indicate that any increase in DA release is counteracted by DA turnover or impaired reuptake, potentially reflecting altered DAT function reported in DYT1 models [5]. Overall, our findings suggest that PV+ FSI inhibition does not acutely affect dopaminergic output in the striatum and that dopaminergic signaling appears relatively unaltered under baseline, non-challenged conditions. This preservation may reflect compensatory stabilization of DA homeostasis despite deficits in other striatal microcircuits.

### 3.4. Serotonergic Signaling: Reduced 5-HIAA Levels in DYT1 KI Mice

Although serotonin (5-HT) was below the limit of detection in all samples, its metabolite 5-hydroxyindoleacetic acid (5-HIAA) was reliably quantified and revealed a genotype-dependent reduction. DYT1 KI mice showed significantly lower 5-HIAA concentrations during post-stimulation, with a trend towards reduced levels during stimulation, indicating an impaired serotonergic response to striatal PV+ FSI inhibition.

Under physiological conditions, inhibition of PV+ FSI is expected to disinhibit SPN, increasing striatal output activity and probably triggering compensatory neuromodulatory mechanisms aimed at stabilizing striatal excitability. These mechanisms include phasic 5-HT release from raphe-striatal terminals [54,55]. Because extracellular 5-HT is rapidly cleared via the serotonin transporter (SERT), microdialysis is often unable to detect transient 5-HT changes directly. For this reason, 5-HIAA serves as a stable marker for serotonergic turnover [56]. Analysis of 5-HIAA dynamics revealed a moderate decline during stimulation, followed by a post-stimulation rebound in both genotypes. This pattern suggests a temporal discrepancy: rapid 5-HT release and reuptake temporarily store serotonin, delaying its metabolism to 5-HIAA. The genotypic difference was most notable in the post-stimulation period, with WT mice exhibiting a more pronounced post-stimulation overshoot compared to DYT1 KI mice. Our findings of reduced 5-HIAA levels in DYT1 KI mice align with clinical reports of impaired serotonergic signaling in human patients with dystonia [57] and support the hypothesis that serotonergic dysfunction may contribute to abnormal motor network excitability [58]. While the technical challenges in measuring synaptic 5-HT [25] preclude definitive conclusions about release dynamics, the robust deficit in 5-HIAA provides evidence of a compromised serotonergic system in dystonia.

### 3.5. Cholinergic Alterations and Exploratory Insights into Iso-Acetylcholine

Interpretation of extracellular acetylcholine (ACh) dynamics was limited by the small sample size of quantifiable ACh concentrations (WT *n* = 4, DYT1 KI *n* = 3). While ACh levels remained relatively stable in WT mice, they appeared lower in DYT1 KI mice during and after PV+ FSI inhibition, though without reaching statistical significance, likely due to low statistical power.

In contrast, choline levels were robustly quantified and showed a significant decrease from baseline to post-stimulation in both genotypes, suggesting preserved acetylcholinesterase (AChE) activity. Since choline is the primary metabolite of ACh, its extracellular concentration reflects a balance between ACh degradation and choline reuptake [59]. The observed reduction in choline, despite unchanged or decreased ACh, could indicate a complex shift in cholinergic signaling. This may reflect an initial increase in cholinergic turnover, followed by net depletion or a reduction in ACh release, leading to less substrate or degradation. Under physiological conditions, inhibition of PV+ FSI is expected to enhance cholinergic activity indirectly by altering local striatal inhibition and network dynamics [33,60,61]. PV+ FSI and cholinergic interneurons (ChIN) interact in an asymmetric manner. ChIN have been shown to directly excite PV+ FSI, where, in contrast, PV+ FSI do not directly inhibit ChIN [62,63]. Consequently, during optogenetic inhibition of PV+ FSI, any alterations in cholinergic output are indirect, arising from modified network dynamics [16]. The blunted ACh response in DYT1 KI mice suggests that an altered cholinergic phenotype may underlie this aberrant response profile [12,64,65]. Future studies specifically optimized for cholinergic measurements may benefit from extended sampling strategies or targeted analytical approaches to increase the number of quantifiable ACh samples and improve statistical power for detecting subtle cholinergic effects.

The isobaric compound iso-acetylcholine (isoACh), also known as gamma-butyrobetaine (GBB), was reliably separated from ACh by chromatographic separation [25]. Although no significant effects were found on isoACh levels, its presence in striatal dialysates indicates it may play a role in neuromodulation. The physiological role of isoACh is still unclear. However, it has been suggested that isoACh might interact with both GABAergic and cholinergic systems [66,67]. Its response to PV+ FSI inhibition should be subtle and indirect, affecting network metabolism rather than direct release dynamics. The isobaric mass with ACh reinforces the importance of chromatographic separation to avoid misinterpretation of ACh measurements [68].

### 3.6. Strengths and Limitations

This study advances the methodology for investigating circuit-level neurotransmitter dynamics by integrating optogenetics with microdialysis (optodialysis) in awake, freely moving mice, presenting physiological circuit dynamics [19,25]. Key strengths include the spatially precise alignment of optical fibers and microdialysis probes (ensuring sampling within the optical stimulation range), the use of low perfusion rates (0.5 µL/min, enhancing analyte recovery), and in vitro recovery correction to account for inter-probe variability. Standardized surgical procedures, extended post-surgical recovery periods, and exclusion of animals with unstable baseline recordings further ensured data reliability and reproducibility.

Despite these methodological advances, some important limitations must be acknowledged. First, although PV+ FSI inhibition robustly reduces neuronal activity [19], optodialysis revealed selective effects on extracellular GABA and adenosine levels, while other analytes remained largely unaffected. This likely reflects limitations of microdialysis, including restricted temporal and spatial resolution [69] and incomplete equilibrium across the dialysis membrane, which can obscure fast, phasic, or highly localized neurochemical events.

Second, reliable quantification of serotonin (5-HT), homovanillic acid (HVA), and acetylcholine (ACh) was not achievable in the majority of samples, as concentrations fell below the limit of quantification (0.07 ng/mL, 17.1 ng/mL, and 0.03 ng/mL, respectively) [25,70,71]. The use of low perfusion rates (0.5 µL/min) was a deliberate methodological choice to maximize analyte recovery, particularly for low-abundance neurotransmitters. However, it inherently extends the sampling interval required per fraction, thereby reducing temporal resolution. Nevertheless, median recovery rates of 15% were determined, confirming adequate membrane permeability despite the low perfusion rate. The presence of diverse chemical structures, such as neurotransmitters and neuromodulators, gave rise to a range of technical impediments in the analytical process [70]. The analytical method prioritized chromatographic specificity and endogenous signaling reliability over maximum analyte sensitivity, as exemplified by the chromatographic resolution of ACh from iso-ACh.

Third, the additional optical implant introduced variability through changes in probe placement, localized tissue damage, and alteration in perfusion and extracellular space [71], accountable for the greater baseline variability compared to the work from Becker et al. [25]. While microdialysis captures tonic, extrasynaptic neurotransmitter dynamics with high neurochemical specificity, it does not resolve rapid phasic events occurring on a millisecond timescale [72]. The present study was designed to exploit precisely this strength: characterizing stimulus-evoked changes in extrasynaptic neurotransmitter tone rather than synaptic release kinetics and should therefore be interpreted within this methodological scope. Sample variability in optodialysis experiments is inherently higher than in conventional microdialysis, owing to several compounding factors. Most notably, the necessary handling of animals prior to each experiment constitutes an unavoidable stressor, causing stress-related neurotransmitter perturbations during baseline periods. Behavioral responses and light-induced heating [73] further increase sample variability, lowering sensitivity for low-abundance neurotransmitters compared to traditional microdialysis.

Consequently, the values designated as baseline reflecting in the present study are more accurately interpreted as pre-stimulation values, reflecting a post-handling neurochemical state under a minimum of light rather than true resting conditions. This should be considered when comparing baseline concentrations with those reported in conventional microdialysis studies.

Also, an attempt was made to simultaneously record locomotor behavior using automated tracking software, as described in Schulz et al. [19]. However, the microdialysis setup—specifically the two-arm swivel and transparent plastic cylinders—caused extensive light reflections and movement artefacts, rendering behavioral data unreliable. Consequently, no concurrent behavioural correlates could be established in the present study. It is recommended that future studies on optodialysis employ reflection-minimized chambers (e.g., infrared-based tracking in opaque enclosures) in order to facilitate meaningful correlations between behaviour and neurochemistry.

These limitations notwithstanding, the present study establishes optodialysis as a viable approach for circuit-specific neurochemical profiling in vivo, and the identified methodological constraints provide a clear roadmap for technical refinement in future investigations.

### 3.7. Further Perspectives

Despite these limitations, the genotype-specific attenuation of GABA and ADE responses supports the concepts of impaired inhibitory control within the striatal microcircuit in DYT1 dystonia [5]. The delayed decline in ADE levels in DYT1 KI mice suggests disrupted neuromodulatory adaptation, potentially reflecting altered A1/A2A receptor-mediated neuromodulation [47]. Further studies should examine whether A1/A2 receptor expression or coupling is altered in DYT1 KI mice. Similarly, the functional interplay between PV+ FSI and striatal cholinergic interneurons has not been directly examined in the context of DYT1 dystonia. Becker et al. [25] were the first to chromatographically resolve ACh from its isobaric isomer isoACh (gamma-butyrobetaine) in striatal microdialysates. This analytical distinction was not achieved in previous microdialysis studies, where the isoACh signal may have contributed to reported ACh concentrations. This unique methodological capability revealed a non-significant trend toward reduced post-stimulation isoACh levels in DYT1 KI mice. This raises the question of whether isoACh is neurobiologically active or a metabolic byproduct in DYT1 dystonia. The physiological role, biosynthetic origin, and potential pathophysiological relevance of isoACh in DYT1 dystonia therefore remain poorly characterized. This represents a promising and tractable avenue for future neurochemical investigation.

## 4. Materials and Methods

### 4.1. Animals

Animal care and all experiments were in accordance with the European guidelines (Directive 2010/63/EU) and the German Welfare Agency (ethical permission number TVV 08/22). Groups of 6-month-old (mean ± SD: 197.52 ± 7.98 days) heterozygous DYT1 KI mice (TOR1A+/ΔGAG, DYT1 KI *n* = 12) and wildtype littermates (WT *n* = 11) of both sexes were used. Both sexes were included with approximately equal distribution (Table S3). All animals were on a C57BL/6J background and expressed eNpHR3.0/EYFP in parvalbuminergic cells.

The establishment of the mouseline is described in detail in Schulz et al. [19]. In brief, homozygous mice expressing Cre-Recombinase in parvalbuminergic cells (Pvalbtm1.1(cre)Aibs, The Jackson Laboratory, Bar Harbor, ME, USA) were crossed with homozygous mice expressing an improved halorhodopsin eNpHR3.0/EYFP (Ai39(RCL NpHR3.0/EYFP, The Jackson Laboratory, Bar Harbor, ME, USA). The resulting offspring expressing Cre-mediated eNpHR3.0/EYFP in parvalbuminergic cells were then crossed with heterozygous DYT1 KI mice [4,19].

Genotyping was performed at weaning (postnatal day 21), using polymerase chain reaction (PCR) amplification analysis of ear tissue samples (PuReTaq Ready-To-Go Beads, GE Healthcare). Mice were housed under controlled conditions (24 ± 2 °C, ~40% relative humidity, 12 h light/dark cycle) in conventional Makrolon cages (type III) with bedding and nesting material. Food (standard diet for mice, Altromin, Lage, Germany) and water were available ad libitum. All experiments were carried out during the dark phase in a special experimental room under identical controlled conditions.

### 4.2. Stereotaxic Surgery

Three days prior to surgery, animals were single-housed in high Makrolon cages. Before surgery, body weight and health status were assessed. Anesthesia was induced with 4% isoflurane (CP-Pharma, Burgdorf, Germany) at a flow rate of 400 mL/min and maintained at 2.2% isoflurane at 220 mL/min with 22% O_2_ (Univentor 1200 Anesthesia Unit, Univentor, Zejtun ZTN 3000, Malta). After fixation in a stereotaxic frame (Stoelting, Wood Dale, IL, USA), eye ointment was applied and mice were placed on an integrated heating mat (Braintree scientific, Braintree, MA, USA) with continuous temperature monitoring via rectal probe (ThermoWorks, American Fork, UT, USA).

**Figure 8:**
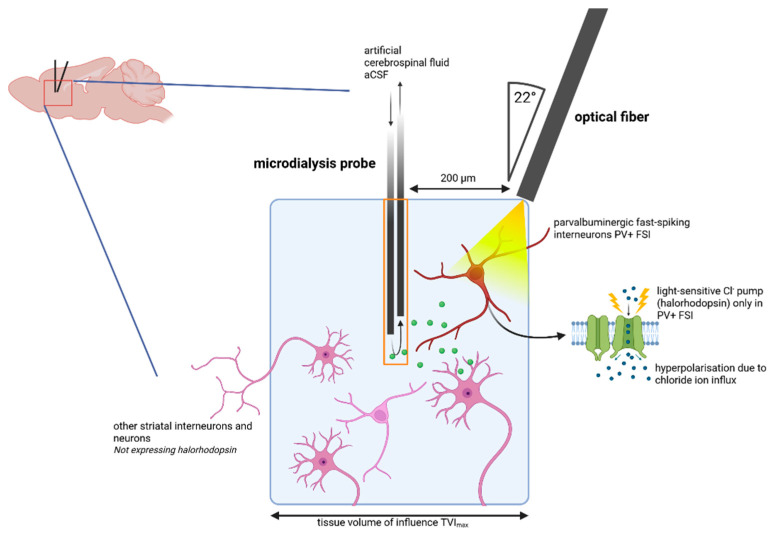
Schematic illustration of striatal implantation. The microdialysis (MD) probe was implanted in the dorsal striatum to measure neurotransmitter concentrations within a tissue volume under influence (TVI) with a radius of 600 µm [75]. An optical fiber was placed within ≤200 µm distance of the MD probe to enable light delivery to striatal parvalbuminergic fast-spiking interneurons (PV+ FSI, red). Yellow light (595 nm) activated the light-driven chloride pump halorhodopsin (eNpHR3.0, green), resulting in neuronal inhibition. Optical irradiance (~1.2 mW/mm^2^) reached a tissue depth of up to 275 µm, enabling in vivo microdialysis during optogenetic modulation. Green dots represent neurotransmitters. Created with BioRender.com.

After securing the head with ear bars, 0.1% bupivacaine (Jenapharm, Jena, Germany) was applied subcutaneously along the incision line to provide peri- and postoperative analgesia. A midline skin incision was made to expose the skull and the cranial sutures with the reference points bregma and lambda. Using Paxinos and Franklin [74] coordinates, a microdialysis (MD) guide cannula (CMA 7, diameter 0.24 mm, length 1 mm, CMA Microdialysis AB, Kist, Sweden) was unilaterally implanted in the dorsal striatum (relative to bregma, in mm: anteroposterior +0.8, mediolateral +1.9, dorsoventral −2.4). An optogenetic LED fiber cannula (core diameter 200 µm, outer diameter 225 µm, length 2.6 mm, numeric aperture 0.66, Prizmatix, Holon, Israel) was implanted ipsilaterally at a 22° angle (anteroposterior −0.7, mediolateral +1.9, dorsoventral −2.6). The distance between the MD guide cannula and fiber tip was <0.2 mm to ensure sufficient irradiance for eNpHR3.0 activation and simultaneous MD sampling within an optogenetic inhibition area of PV+ FSI [75] (Figure 8). Both implants and three additional stainless steel holding screws were secured with UV Adhesive (Heliobond, ivoclar vivadent, Ellwangen, Germany) and covered in dental acrylic cement (Paladur, Heraeus Kulzer, Hanau, Germany) mixed with black food colorant. A black plastic sleeve covered the optical fiber to prevent light leakage during optogenetic stimulation. After surgery, mice recovered for 5–7 days with daily health monitoring.

### 4.3. Optodialysis Procedure

Optodialysis, combining microdialysis with optogenetic stimulation [19,24], was preceded by in vitro recovery testing using a standard neurotransmitter mixture (15 ng/mL in artificial cerebrospinal fluid, aCSF, µdialysis, Stockholm, Sweden) to calibrate each MD probe. Probes (CMA7, 1 mm length, molecular cut-off 6 kDa) were perfused with aCSF (NaCl 147, KCl 2.7, CaCl_2_ 1.2, MgCl_2_ 0.85) at a flow rate of 0.5 µL/min. Three samples were collected using a CMA/420 microinjection pump (Carnegie Medicine, CMA Microdialysis AB, Kista, Sweden) and immediately stored at −80 °C.

For in vivo sampling, the MD probe was lowered gently through the implanted MD guide cannula under continuous perfusion with aCSF at 0.5 µL/min. Then, aCSF flow was interrupted and mice were connected with a counterbalancing arm carrying a two-channel swivel (CMA 120, Carnegie Medicine, CMA Microdialysis AB, Kista, Sweden), allowing free movement. After overnight habituation in a plastic cylinder with bedding, food, and water gel, mice were connected between the implanted optogenetic fiber (Prizmatix, Holon, Israel) and the fiber patch cords via sleeves. Then, after discarding dead volume, 9 consecutive dialysate samples (36 min each) were collected at 0.5 µL/min aCSF, in glass vials coated with evaporated stabilizers (0.25 mM ascorbic acid and 0.1 M perchloric acid) using a refrigerated fraction collector at 6 °C (CMA/170, CMA Microdialysis AB, Kista, Sweden). Three samples each were collected for baseline (light off), stimulation (light on), and post-stimulation (light off). Samples were immediately frozen and stored at −80 °C until LC MS/MS analysis.

**Figure 9:**
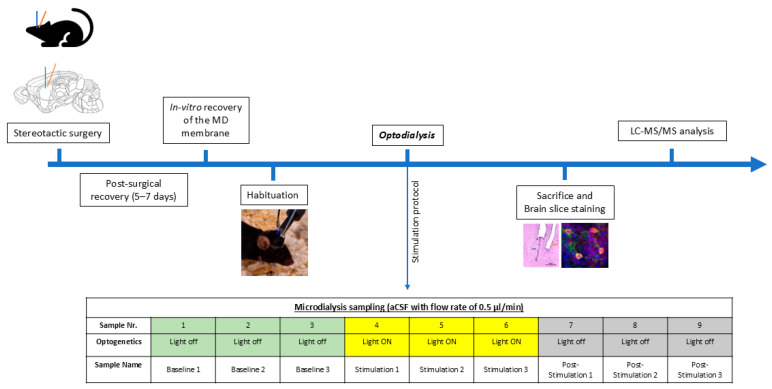
Experimental design. After unilateral implantation of the microdialysis guide cannula and optical fiber, mice recovered for 5–7 days. In vitro recovery was conducted to normalize the MD membrane. Mice habituated overnight in a plastic cylinder. The day after, sampling started according to the stimulation and sampling protocol. After the experiment, brains were collected for immunohistochemical labeling and Nissl staining. Samples were analyzed using liquid chromatography-tandem mass spectrometry (LC-MS/MS).

**Figure 10:**
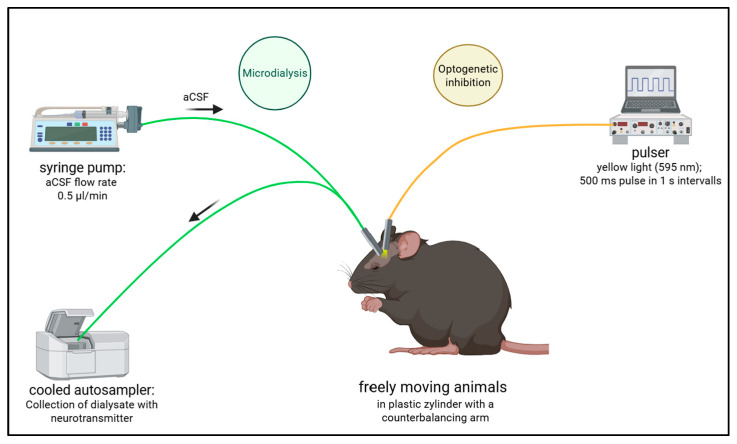
Optodialysis setup. During the optodialysis experiment, freely moving mice were connected to the MD system for sampling (green), and the fiber patch cords for optogenetic inhibition of PV+ FSI (yellow). The MD set-up included a syringe pump delivering aCSF at 0.5 µL/min. The optogenetic fiber is connected to a pulser, which sets the light pulses according to a specific stimulation protocol. Created with BioRender.com.

For the light stimulation period, intensities of the LED fiber were measured prior to the surgical implantation and set at 2–3 mW, using a 595 nm yellow LED light source suitable for eNpHR3.0 activation, as described [19]. Pulse duration and interval length were set at a 500 ms light pulse with 1 s intervals, controlled by a pulser (Prizmatix Pulser Ver. 2.3, Prizmatix, Holon, Israel). These parameters were based on previous studies using improved halorhodopsin eNpHR3.0 [76,77] and previous experimental findings [19]. Figure 9 illustrates the experimental design in chronological order, and a close look at the setup is demonstrated in Figure 10.

### 4.4. LC-MS/MS Analysis

MD samples were analyzed using LC MS/MS, as previously described [24,25]. Briefly, 15 µL of each MD sample was mixed with 1.5 µL of internal standard mixture, vortexed, and placed in a cooled autosampler. Chromatographic separation (UHPLC Biphenyl RP-18e pre-column (3.0 mm ID), Phenomenex (Aschaffenburg, Germany)) of GABA, DA, 3-MT, 5 HIAA, ACh, choline, isoACh, and ADE was performed on a 1290 Infinity II LC system from Agilent (Duisburg, Germany) coupled to a QTRAP LC-MS/MS 5500 from SCIEX (Framingham, MA, USA) using positive electrospray ionization (ESI) mode combined with multiple reaction monitoring (MRM) detection. Quantitative analysis was carried out using Analyst 1.7 software (SCIEX, Montreal, Quebec, Canada).

The lower limits of quantification (LLOQ) with a signal-to-noise ratio of 10:1 were as follows: 0.025 ng/mL for GABA, 0.25 ng/mL for DA, 0.75 ng/mL for 3-MT, 0.5 ng/mL for 5-HIAA, 0.015 ng/mL for choline, 0.1 ng/mL for isoACh, and 0.07 ng/mL for adenosine [25]. Samples from each individual mouse were analyzed within the same analytical batch.

Mean inter-batch coefficients of variation across all 11 batches were 7.8% for GABA, 9.8% for DA, 8.0% for 3-MT, 12.6% for 5-HIAA, 13.8% for isoACh, 7.3% for choline, and 20.8% for adenosine. With regard to variance, a stable precision of the measurement method has proven itself. Analyte levels were standardized based on their respective in vitro recovery rates for each membrane used.

### 4.5. Brain Slice Staining and Image Acquisition

After the experiment, mice were euthanized (pentobarbital, >400 mg/kg, intraperitoneal injection) and transcardially perfused with 0.1 M phosphate-buffered saline (PBS) and 4% paraformaldehyde (PFA). Brains were removed and fixed in 4% PFA solution for 24 h, followed by incubation in increasing PBS-sucrose solutions (10–20–30%), each for 24 h. Brains were then frozen on dry ice and stored at −80 °C until sectioning into 40 µm sagittal slices at 20 °C using a cryostat (Hypax C 50, Zeiss, Jena, Germany). Sections were stored at 20 °C in cryoprotectant solution.

Brain slices were used to validate specific expression of eNpHR3.0 in parvalbuminergic cells via immunohistochemical (IHC) labeling, as previously described in detail in Schulz et al. [19]. Briefly, striatal slices were washed and blocked in 50 mM tris buffered saline (TBS, blocking solution: TBS, 10% normal donkey serum, 0.5% Triton X) before overnight incubation at 4 °C with primary antibody against parvalbumin (Rabbit anti-parvalbumin, 1:2000, Synaptic Systems). The following day, slices were washed in 50 mM tris buffered saline (TBS) and incubated with the secondary antibody (Donkey anti-rabbit, 1:800, Alexa Fluor 594, Jackson ImmunoResearch Labs) for 1 h. Finally, slices were washed in TBS, transferred to glass slides, cover-slipped with VectaShield H-100 with DAPI (Vector Laboratories, Newark, CA, USA), and stored at 4 °C.

Images were acquired using a Zeiss Axioskop microscope coupled to Zeiss Axiocam 506 mono camera (Carl Zeiss Microscopy GmbH, Oberkochen, Germany), a Retiga 2000R CLR-12 color digital camera (QImaging, Surrey, British Columbia, Canada), and the Stereo Investigator software (Version 2023.2.1, MBF Bioscience, Williston, ND, USA). Implant localization, including distance and angulation, was verified postmortem in sagittal brain slices for all mice using Nissl staining.

### 4.6. Statistics

Statistical analysis and data visualization were performed using SigmaPlot16.0 (Systat, Chicago, IL, USA). Results from the LC MS/MS analysis were processed by calculating mean values for each stimulation period. Since striatal extracellular baseline values decrease over time and reach equilibrium [24], a stable baseline could be derived from the second MD sample onward. Hence, neurotransmitter levels were averaged across two baseline samples (2nd and 3rd), three stimulation samples, and three post-stimulation samples per mouse. The in vitro recovery rate depends on the MD setup and differs between MD membranes of the same charge. Therefore, it is necessary to normalize extracellular neurotransmitter concentrations:

Recovery rate = dialysate concentration/standard concentration(1)

All statistics were performed on data normalized to the in vitro recovery rate of the used membrane for each animal. A two-way repeated measures analysis of variance (Two-way RM ANOVA) was used to assess the effects of stimulation (baseline, light stimulation, post stimulation) and genotype (DYT1 KI, WT). This allows the identification of genotype effects (as a between-subject factor), stimulation effects (as a within-subject factor), and genotype x stimulation interaction effects. Post hoc multiple comparisons were performed using Holm Sidak. Statistical outliers were detected using Grubb’s test and removed from the data set. Concentrations of neurotransmitters exceeding the limit of detection (LOD) but falling below the LLOQ were excluded from statistical analysis.

Neurotransmitter ratios were calculated as the concentration of the primary substance relative to its metabolite:

Ratio = metabolite concentration/originating substance concentration (2)

Additionally, two-way RM ANOVA was also used to assess genotype and stimulation effects as well as possible interaction.

Significance was assigned at *p* < 0.05.

#### Power Analysis

The number of animals required was determined using an a priori power analysis (G*Power 3.1). Based on an expected effect size of Cohen’s f = 0.6 (F-test, ANOVA), a significance level of α = 0.05, and a statistical power of 1 − β = 0.8, the required sample size was *n* = 12 animals per genotype. As the animals served as their own control group for extracellular neurotransmitter analysis, the study design increased the statistical power for within-subject comparisons. Although the sample size was not reached due to technical limitations (actual sample: DYT1 KI *n* = 10, WT *n* = 9), a post hoc power analysis to ensure statistical validity for the two-way RM ANOVA showed sufficient power (within factor 1 − β = 0.99; between-factors: 1 − β = 0.85; interactions: 1 − β = 0.99). Therefore, the number of animals was not further increased, which is in accordance with the 3R principles (Replace, Reduce, Refine).

## 5. Conclusions

Optogenetic inhibition of PV+ FSI leads to genotype-specific alterations in striatal neurotransmitter dynamics in a DYT1 KI mouse model. Most notably, GABA and ADE responses were selectively impaired in DYT1 mice, while dopaminergic and cholinergic systems remained largely unaffected, indicating a circuit-level specificity that would not have been detectable under baseline conditions alone. These findings indicate impaired inhibitory and neuromodulatory control in DYT1 mice, consistent with compensatory circuit adaptations arising from TorsinA dysfunction. The selective disruption of GABA and ADE signaling, in the absence of dopaminergic or cholinergic alterations, points to PV+ FSI as a nodal element in the striatal microcircuit whose dysfunction may contribute to the latent neurochemical imbalances underlying DYT1 dystonia.

The present study demonstrates the feasibility of optodialysis—the combined application of in vivo optogenetics and microdialysis for simultaneously monitoring multiple extracellular neurotransmitters under resting and circuit-perturbed conditions in freely moving mice. This approach establishes a reproducible methodological framework applicable to a broad range of genetic movement disorder models beyond DYT1 dystonia.

## Figures and Tables

**Table 1:** Extracellular concentrations (ng/mL, mean ± SEM) of neurotransmitters and metabolites across stimulation conditions in WT and DYT1 KI mice. Concentrations of GABA, dopamine (DA), 3-methoxytyramine (3-MT), acetylcholine (ACh), iso-acetylcholine (isoACh), choline, adenosine (ADE), and 5-hydroxyindolacetic acid (5-HIAA) across three conditions: baseline (light off), during optogenetic inhibition of PV+ interneurons (stimulation, light on), and the post-stimulation period for WT and DYT1 KI mice. Values represent the average of three consecutive microdialysis (MD) samples, normalized to the individual in vitro recovery of the MD membrane. Statistical comparisons were made for: (1) baseline vs. stimulation, (2) baseline vs. post-stimulation, and (3) stimulation vs. post-stimulation. Significant differences are marked as follows: * within WT, ^#^ within DYT1 KI, ^G^ between genotypes.

	GABA ^(1), (2)^	ACh	IsoACh	Cholin ^(1), (3)^	DA	3-MT	5-HIAA	ADE ^(1), (2)^
	WT (*n* = 8)	WT (*n* = 4)	WT (*n* = 9)	WT (*n* = 8)	WT (*n* = 9)	WT (*n* = 9)	WT (*n* = 9)	WT (*n* = 9)
	DYT1 (*n* = 9)	DYT1 (*n* = 3)	DYT1 (*n* = 9)	DYT1 (*n* = 9)	DYT1 (*n* = 9)	DYT1 (*n* = 10)	DYT1 (*n* = 9)	DYT1 (*n* = 9)
*Baseline*								
WT	10.25 ± 2.23 *	0.48 ± 0.04	7.74 ± 0.99	153.36 ± 25.64	3.37 ± 0.74	7.15 ± 1.29	150.84 ± 22.28	26.58 ± 7.30 *
DYT1	6.57 ± 0.97	0.49 ± 0.07	6.73 ± 0.97	143.68 ± 23.01	3.19 ± 0.59	6.43 ± 0.59	100.74 ± 15.97	31.96 ± 9.85 ^#^
*Stimulation*								
WT	7.64 ± 1.37 *	0.51 ± 0.10	7.94 ± 1.04	149.08 ± 29.73	3.33 ± 0.71	7.52 ± 1.29	144.65 ± 18.79	14.90 ± 4.63 *
DYT1	5.82 ± 0.82	0.37 ± 0.04	5.98 ± 0.67	128.33 ± 22.85	2.54 ± 0.23	6.21 ± 0.51	89.70 ± 18.10	23.64 ± 7.84
*Post-Stim*								
WT	7.98 ± 1.44 *	0.52 ± 0.07	7.34 ± 0.86	127.51 ± 30.52	6.50 ± 0.76	7.77 ± 1.24	159.15 ± 21.91 ^G^	11.23 ± 2.79 *
DYT1	6.01 ± 1.04	0.38 ± 0.02	5.77 ± 0.62	115.50 ± 23.28	2.77 ± 0.17	6.34 ± 0.37	91.99 ± 15.19 ^G^	18.54 ± 5.25 ^#^

## Data Availability

The original contributions presented in this study are included in the article/Supplementary Materials. Further inquiries can be directed to the corresponding author.

## Supplementary Materials

The following supporting information can be downloaded at: https://www.mdpi.com/article/10.3390/ijms27104530/s1.

## Abbreviations

The following abbreviations are used in this manuscript:

3-MT	3-methoxytyramine
5-HIAA	5-hydroxyindoleacetic acid
ACh	acetylcholine
ADE	adenosine
DA	dopamine
DYT1 KI	knock-in mouse model for DYT1 dystonia
GABA	γ-aminobutyric acid
isoACh	γ-butyrobetaine (former iso-acetylcholine), isobaric compound of ACh
MD	microdialysis
PV+ FSI	Parvalbumin-positive fast-spiking interneurons
