# Supplementary material for: Optogenetic Inhibition of Striatal Parvalbuminergic Interneurons Unmasks Impaired GABA and Adenosine Signaling in DYT1 Knock-In Mice
Source: Int J Mol Sci. 2026 May 18;27(10):4530. doi: 10.3390/ijms27104530 (PMC13207817; doi:10.3390/ijms27104530)
Supplement: Supplementary file 1 [file ijms-27-04530-s001.zip › Supplementary Table-S1.pdf]

Table S1. Technical and biological replicates showing baseline, stimulation, and post-stimulation concentrations of quantifiable neurotransmitters and neuromodulators for each **DYT1 knock-in mouse** (n = 10). Reported concentrations (ng/ml) are standardized to the in vitro recovery rate of the membrane used per animal. For each stimulation period, corresponding mean values (M) and standard deviations (SD) are provided (mean ± SD).

Animal	Parameter	Sample	DA	ACh	isoACh	5-HIAA	3-MT	GABA	Cholin	ADE
		1.	[a]4,45	<LLOQ	[a]102,99	[a]2131,29	[a]19,34	[a]178,01	[a]671,31	[a]538,72
		2.	[b]8,52	<LLOQ	[b]23,90	[b]566,49	[b]11,41	[b]33,63	300,04	[b]88,25
	Baseline	3.	[b]9,71	<LLOQ	13,52	[b]319,93	7,88	13,30	200,71	22,37
		SD	2,76		0	[c]174,35	0	0	70,24	0
		**M**	**[c]7,56**		**13,52**	**[c]290,99**	**7,88**	**13,30**	**250,38**	**22,37**
		1.	[a]11,00	<LLOQ	12,42	[b]289,74	10,49	[b]19,49	202,08	24,10
		2.	[a]19,40	<LLOQ	10,44	[b]307,39	[b]30,16	[b]26,03	235,65	23,62
**1**	Stimulation	3.	[b]8,57	<LLOQ	8,70	[b]201,06	8,08	10,69	164,06	21,32
		SD	5,68		1,86	0	1,70	0	35,82	1,49
		**M**	**[c]12,99**		**10,52**	**[c]201,06**	**9,29**	**10,69**	**200,60**	**23,01**
		1.	[b]7,71	<LLOQ	9,77	[b]214,52	6,79	12,81	167,49	22,47
		2.	[b]6,83	<LLOQ	9,38	[b]238,67	8,45	8,72	183,93	24,58
	Post-Stimulation	3.	[b]6,61	<LLOQ	9,77	[b]192,70	6,34	10,91	174,68	22,57
		SD	0,58		0,22	22,99	1,11	2,04	8,24	1,19
		**M**	**[c]7,05**		**9,64**	**[c]215,30**	**7,19**	**10,81**	**175,36**	**23,21**
		1.	1,44	<LLOQ	6,64	[a]120,51	7,05	[a]10,04	103,90	[a]76,85
		2.	1,88	<LLOQ	4,39	89,58	6,09	3,80	88,19	5,17
	Baseline	3.	3,46	<LLOQ	6,64	56,24	8,31	4,50	115,12	4,94
		SD	1,06		1,30	23,58	1,11	0,49	13,53	0,17
		**M**	**2,26**		**5,89**	**72,91**	**7,15**	**4,15**	**102,40**	**5,06**
		1.	3,02	<LLOQ	4,80	40,57	6,52	5,42	93,34	3,99
		2.	3,46	<LLOQ	5,17	54,63	8,98	5,66	72,87	5,50
**2**	Stimulation	3.	2,88	<LLOQ	5,65	53,83	8,18	2,87	69,44	5,27
		SD	0,30		0,43	7,90	1,26	1,55	12,92	0,81
		**M**	**3,12**		**5,21**	**49,68**	**7,89**	**4,65**	**78,55**	**4,92**
		1.	3,37	<LLOQ	5,14	52,62	8,45	2,64	63,50	5,20
		2.	3,65	<LLOQ	5,37	39,17	9,11	3,13	51,22	5,17
	Post-Stimulation	3.	3,99	0,75	[b]25,82	47,40	7,85	[b]20,17	146,54	5,24
		SD	0,31		0,16	6,78	0,63	0,35	51,85	0,03
		**M**	**3,67**		**5,26**	**46,40**	**8,47**	**2,89**	**87,09**	**5,20**
		1.	1,27	<LLOQ	5,71	64,57	4,34	[a]11,64	163,86	9,73
		2.	1,20	<LLOQ	5,63	88,87	4,78	5,12	175,92	6,51
	Baseline	3.	1,46	<LLOQ	6,13	84,35	4,73	5,94	165,10	5,09
		SD	0,13		0,27	12,93	0,24	0,58	6,63	2,38
**3**		**M**	**1,31**		**5,82**	**79,26**	**4,62**	**5,53**	**168,29**	**7,11**
		1.	1,63	<LLOQ	5,76	93,03	5,11	3,35	198,18	5,20
		2.	1,44	<LLOQ	6,66	97,20	5,38	5,55	198,49	5,82
	Stimulation	3.	1,66	<LLOQ	6,11	78,11	6,03	5,66	222,60	5,22
		SD	0,12		0,46	10,04	0,47	1,31	14,01	0,35

		**M**	**1,58**		**6,18**	**89,45**	**5,51**	**4,85**	**206,42**	**5,41**
	Post-Stimulation	1.	2,39	<LLOQ	7,18	100,67	6,15	5,98	221,37	6,00
		2.	2,57	<LLOQ	7,37	111,08	7,60	8,87	207,14	6,38
		3.	2,78	<LLOQ	6,86	125,32	7,73	6,76	189,52	6,95
		SD	0,19		0,26	12,37	0,88	1,50	15,95	0,48
		**M**	**2,58**		**7,13**	**112,36**	**7,16**	**7,20**	**206,01**	**6,44**
**4**	Baseline	1.	7,85	0,77	[a]30,80	[a]91,29	[a]23,03	[a]129,25	[a]334,73	[a]471,99
		2.	4,22	<LLOQ	9,47	54,11	10,95	[b]22,96	203,58	[b]156,42
		3.	3,51	<LLOQ	5,73	35,02	8,68	4,54	158,75	10,52
		SD	2,33		2,64	13,50	1,61	0	31,70	0
		**M**	**5,19**		**7,60**	**44,56**	**9,82**	**4,54**	**181,17**	**10,52**
	Stimulation	1.	3,29	<LLOQ	5,88	38,17	7,70	4,87	131,54	8,34
		2.	3,11	<LLOQ	5,35	31,20	7,27	2,70	136,83	7,74
		3.	3,88	<LLOQ	5,25	34,85	8,91	3,85	111,19	7,58
		SD	0,40		0,34	3,49	0,85	1,09	13,54	0,40
		**M**	**3,43**		**5,49**	**34,74**	**7,96**	**3,80**	**126,52**	**7,88**
	Post-Stimulation	1.	3,07	<LLOQ	4,18	28,55	6,94	3,28	96,70	6,59
		2.	3,23	<LLOQ	4,77	21,74	6,70	6,08	103,16	7,63
		3.	3,30	<LLOQ	4,23	24,07	7,49	6,45	92,39	6,81
		SD	0,12		0,33	3,46	0,41	1,73	5,42	0,55
		**M**	**3,20**		**4,40**	**24,79**	**7,04**	**5,27**	**97,42**	**7,01**
**5**	Baseline	1.	[a]8,78	0,74	[a]20,11	[a]346,85	[a]13,86	[a]103,54	[a]423,65	[a]940,44
		2.	2,76	0,34	6,99	211,97	6,21	8,36	224,83	42,79
		3.	2,88	0,38	6,61	168,98	6,10	7,62	247,90	3,69
		SD	0,09	0,22	0,27	30,40	0,08	0,52	16,31	27,65
		**M**	**2,82**	**0,49**	**6,80**	**190,48**	**6,16**	**7,99**	**236,37**	**23,24**
	Stimulation	1.	2,17	0,33	5,87	216,41	5,88	6,32	217,28	0,67
		2.	1,75	0,37	6,48	[b]82,71	4,16	7,77	211,40	10,45
		3.	1,88	0,35	6,11	179,36	4,75	9,54	207,21	2,00
		SD	0,22	0,02	0,31	26,20	0,88	1,61	5,06	5,31
		**M**	**1,93**	**0,35**	**6,15**	**197,88**	**4,93**	**7,88**	**211,96**	**4,37**
	Post-Stimulation	1.	1,99	0,39	6,26	117,25	5,72	13,76	225,67	2,63
		2.	2,10	0,44	6,53	131,92	6,00	10,58	224,41	3,40
		3.	2,24	0,42	5,83	186,77	5,61	8,06	203,02	3,72
		SD	0,12	0,03	0,36	36,64	0,20	2,85	12,73	0,56
		**M**	**2,11**	**0,42**	**6,21**	**145,31**	**5,78**	**10,80**	**217,70**	**3,25**
**6**	Baseline	1.	2,71	0,35	[a]11,53	143,42	8,05	9,87	86,01	[a]77,42
		2.	2,33	0,09	6,14	105,09	4,75	7,23	58,18	17,42
		3.	2,72	0,12	7,43	114,36	6,47	4,15	66,94	15,62
		SD	0,23	0,14	0,91	20,00	1,65	2,86	14,23	1,27
		**M**	**2,59**	**0,19**	**6,78**	**120,96**	**6,42**	**7,08**	**70,38**	**16,52**
	Stimulation	1.	2,49	0,07	5,59	80,36	5,44	3,99	46,62	12,27
		2.	2,83	0,07	5,59	82,84	5,69	3,33	57,55	11,42
		3.	2,57	<LLOQ	5,44	90,87	5,46	3,30	51,22	9,35
		SD	0,18	0,00	0,08	5,49	0,14	0,39	5,49	1,51
		**M**	**2,63**	**0,07**	**5,54**	**84,69**	**5,53**	**3,54**	**51,80**	**11,01**
		1.	2,41	0,85	5,03	70,47	5,19	3,74	54,93	10,34

	Post-Stimulation	2.	2,58	<LLOQ	4,76	63,67	5,12	3,07	46,53	9,30
		3.	2,55	<LLOQ	5,41	87,16	6,48	6,19	45,98	9,02
		SD	0,09		0,33	12,09	0,77	1,64	5,01	0,70
		**M**	**2,51**		**5,07**	**73,77**	**5,59**	**4,33**	**49,15**	**9,55**
**7**	Baseline	1.	5,09	0,03	[a]15,24	[a]200,19	[a]11,96	[a]18,13	[a]142,40	[a]226,29
		2.	4,10	0,06	5,57	96,50	5,85	6,49	76,87	117,14
		3.	3,21	<LLOQ	5,16	110,42	5,02	2,83	74,35	68,57
		SD	0,94	0,02	0,29	9,84	0,59	2,59	1,78	34,34
		**M**	**4,13**	0,05	**5,36**	**103,46**	**5,47**	**4,66**	**75,61**	**92,86**
	Stimulation	1.	3,16	<LLOQ	4,98	40,22	5,24	4,61	62,38	69,14
		2.	3,31	0,06	4,57	49,37	4,80	3,72	66,58	69,14
		3.	3,60	<LLOQ	4,14	[b]117,60	6,04	3,04	33,39	73,71
		SD	0,22		0,42	6,48	0,63	0,79	18,07	2,64
		**M**	**3,35**		**4,56**	**44,80**	**5,36**	**3,79**	**54,12**	**70,67**
	Post-Stimulation	1.	3,37	<LLOQ	4,87	105,48	5,40	2,03	[b]0,31	[b]62,86
		2.	3,48	0,01	3,88	80,79	5,58	4,61	[b]0,14	48,29
		3.	3,18	<LLOQ	3,60	98,30	4,65	0,47	39,00	27,60
		SD	0,16		0,67	12,70	0,50	2,09	0	14,63
		**M**	**3,34**		**4,11**	**94,86**	**5,21**	**2,37**	**39,00**	**37,95**
**8**	Baseline	1.	3,16	0,51	[a]10,93	192,58	7,85	[a]16,07	79,88	[a]188,94
		2.	1,99	0,33	6,76	139,48	5,56	5,92	81,03	59,28
		3.	1,52	0,30	5,78	143,31	5,17	3,14	74,44	52,88
		SD	0,85	0,11	0,69	29,61	1,45	1,97	3,52	4,52
		**M**	**2,23**	**0,38**	**6,27**	**158,46**	**6,19**	**4,53**	**78,45**	**56,08**
	Stimulation	1.	1,95	0,85	8,14	140,12	6,45	[b]137,22	[b]145,65	60,56
		2.	1,81	0,31	6,13	142,68	5,70	5,60	59,53	38,90
		3.	1,86	0,32	6,35	148,43	6,13	5,19	66,12	39,96
		SD	0,07	0,31	1,11	4,26	0,38	0,29	4,66	12,21
		**M**	**1,87**	**0,49**	**6,87**	**143,74**	**6,09**	**5,40**	**62,83**	**46,47**
	Post-Stimulation	1.	2,31	0,32	6,30	144,59	6,80	4,02	53,41	36,08
		2.	2,14	0,35	6,63	153,55	6,24	3,34	31,90	35,23
		3.	2,49	0,52	[b]11,41	166,99	7,92	4,66	[b]3,20	31,77
		SD	0,17	0,11	0,24	11,27	0,86	0,66	15,20	2,28
		**M**	**2,31**	**0,40**	**6,47**	**155,04**	**6,99**	**4,01**	**42,66**	**34,36**
**9**	Baseline	1.	[a]30,61	[a]2,20	[a]39,22	76,52	[a]15,77	[a]154,60	[a]405,22	[a]1041,13
		2.	8,34	0,52	[b]16,49	52,12	8,61	[b]79,66	[b]328,47	[b]484,30
		3.	4,92	0,71	[b]15,84	57,91	6,71	[b]54,82	[b]238,83	[b]346,26
		SD	2,42	0,14	0,71	12,75	1,34	17,56	83,28	97,61
		**M**	**6,63**	**0,61**	**[c]15,99**	**62,18**	**7,66**	**[c]67,24**	**[c]324,18**	**[c]415,28**
	Stimulation	1.	3,25	0,37	[b]13,68	52,95	5,28	10,73	181,12	[b]244,49
		2.	2,75	0,53	[b]14,11	49,22	5,79	12,69	151,04	[b]204,72
		3.	2,63	0,48	[b]12,18	43,02	4,72	7,70	47,58	[b]156,75
		SD	0,33	0,09	1,02	5,02	0,54	2,52	70,05	43,93
		**M**	**2,88**	**0,46**	**[c]13,32**	**48,40**	**5,26**	**[c]10,37**	**126,58**	**[c]201,99**
	Post-Stimulation	1.	3,10	0,30	[b]10,88	47,15	5,20	5,35	[b]0,05	[b]121,66
		2.	2,58	0,37	[b]10,05	51,29	4,88	6,53	[b]0,16	[b]90,66
		3.	2,38	0,35	[b]9,59	60,80	4,84	6,14	[b]0,30	27,14

		SD	0,37	0,04	0,66	7,00	0,20	0,60	[c]0,13	0
		**M**	**2,69**	**0,34**	**[c]10,17**	**53,08**	**4,98**	**[c]6,00**	**[c]0,17**	**[c]27,14**
**10**	Baseline	1.	1,83	<LLOQ	2,45	46,17	2,95	8,51	124,21	70,41
		2.	1,51	<LLOQ	2,53	90,79	2,26	5,89	137,85	51,03
		3.	1,34	<LLOQ	2,59	86,28	3,80	7,71	128,15	40,31
		SD	0,25		0,07	24,56	0,77	1,34	7,02	15,25
		**M**	**1,56**		**2,53**	**74,41**	**3,00**	**7,37**	**130,07**	**53,92**
	Stimulation	1.	1,80	<LLOQ	2,97	82,42	3,58	7,87	140,72	40,44
		2.	2,12	<LLOQ	3,54	118,47	4,61	7,60	165,49	39,94
		3.	2,39	<LLOQ	3,26	141,01	4,77	7,98	180,21	36,64
		SD	0,29		0,28	29,56	0,65	0,19	19,96	2,07
		**M**	**2,10**		**3,26**	**113,97**	**4,32**	**7,82**	**162,14**	**39,00**
	Post-Stimulation	1.	2,13	<LLOQ	3,56	122,34	4,42	7,12	124,21	31,40
		2.	2,56	<LLOQ	3,84	118,47	5,90	4,73	140,00	31,15
		3.	2,81	<LLOQ	3,47	126,20	4,74	7,33	111,28	32,21
		SD	0,34		0,19	3,86	0,78	1,44	14,38	0,55
		**M**	**2,50**		**3,63**	**122,34**	**5,02**	**6,40**	**125,16**	**31,59**

a – The microdialysis system had not yet reached equilibrium. In accordance with Fick's first law of diffusion, the initially steep concentration gradient temporarily altered the effective distribution volume of extracellular neurotransmitters, resulting in artificially elevated baseline values. These values were therefore excluded from the analysis.

b – Values identified as significant outliers by the Grubbs test ($\alpha = 0.05$) were removed from the dataset.

c – Mean values are reported for completeness; however, samples excluded under notes (*a*) and (*b*) were not included in any statistical analyses. If a biological replicate from an animal was excluded, that animal was fully removed from all statistical evaluations.
