# Supplementary material for: Optogenetic Inhibition of Striatal Parvalbuminergic Interneurons Unmasks Impaired GABA and Adenosine Signaling in DYT1 Knock-In Mice
Source: Int J Mol Sci. 2026 May 18;27(10):4530. doi: 10.3390/ijms27104530 (PMC13207817; doi:10.3390/ijms27104530)
Supplement: Supplementary file 1 [file ijms-27-04530-s001.zip › Supplementary Table-S3.pdf]

Table S3. Sex distribution of included animals. Shown are the numbers (n) of male and female mice per genotype (DYT1 KI and WT) and total sample size.

	**DYT1 KI**	**WT**	**Total n**
Male	5	6	11
Female	6	4	10
**Total n**	11	10	**n = 21**
